# Salivary microbiota signatures of periodontitis are associated with CGM-derived short-term glycaemic control in adults with type 1 diabetes: a pilot study

**DOI:** 10.3389/fendo.2026.1794873

**Published:** 2026-03-13

**Authors:** Kevin Munsch, Jiuwen Sun, Thibault Canceill, Paul Slisse, Charlotte Pegouret, Swann Diemer, Pascale Loubières, Sara Laurencin-Dalicieux, Matthieu Minty, Pierre Gourdy, Blandine Tramunt, Remy Burcelin, Vincent Blasco-Baque, Charlotte Thomas

**Affiliations:** 1 Odontology Department, Health Faculty, Toulouse University, Toulouse, France; 2 Odontology Department, Toulouse University Hospital, Toulouse, France; 3 UMR1297 Inserm, Team InCOMM/Intestine ClinicOmics Metabolism & Microbiota, Institute of Metabolic and Cardiovascular Diseases (I2MC), Toulouse, France; 4 UMR1295 Inserm, Team SPHERE, Center for Epidemiology and Research in Population Health (CERPOP), Toulouse University, Toulouse, France; 5 Endocrinology, Diabetology and Nutrition Department, Toulouse University Hospital, Toulouse, France; 6 UMR1297 Inserm, Team AdipoLive, Institute of Metabolic and Cardiovascular Diseases (I2MC), Toulouse, France; 7 Medical Department, Health Faculty, Toulouse University, Toulouse, France

**Keywords:** biomarker, continuous glucose monitoring, oral microbiota, periodontitis, saliva, time above range, type 1 diabetes

## Abstract

**Aims:**

Periodontitis is common in type 1 diabetes (T1D), yet its relationship with short-term glycaemic control remains unclear. Continuous glucose monitoring (CGM) provides complementary metrics to HbA1c, including Time in Range (TIR) and Time Above Range (TAR). We investigated whether periodontitis-associated salivary microbiota signatures are associated with CGM-derived short-term glycaemic metrics in adults with T1D.

**Methods:**

In this pilot cross-sectional study, 24 adults with T1D were classified using the Community Periodontal Index as gingivitis (CPI 1-2; n=12) or periodontitis (CPI 3-4; n=12). Oral indices, HbA1c and CGM metrics were collected. Salivary microbiota was profiled by 16S rRNA gene sequencing. Differential abundance, inferred functional profiling, and multivariate association analyses were used to relate microbial signatures to CGM metrics.

**Results:**

Compared with gingivitis, periodontitis was significantly associated with lower TIR (40.5% *vs* 67.7%; p=0.004) and higher TAR (54.4% *vs* 28.9%; p=0.01), despite similar HbA1c. Gingivitis microbiota was enriched in *Neisseria* and *Lautropia* and associated with higher TIR, whereas *Filifactor* and the *Eubacterium saphenum* group were more found in periodontitis and associated with higher TAR. Predicted functional pathways in periodontitis were led more towards fermentative pathways and amino acid biosynthesis.

**Conclusions:**

In this cohort of adults with T1D, periodontitis was associated with distinct salivary microbiota signatures varied along with CGM-derived short-term glycaemic metrics, despite similar HbA1c. If confirmed in larger longitudinal studies, salivary profiling may be an interesting complement for CGM-based assessment to support integrated periodontal and metabolic follow-up.

## 1 Introduction

Periodontitis is a chronic immuno-inflammatory disease of the oral cavity and has been described as the “sixth complication” of diabetes (1, 2). In type 2 diabetes (T2D), the relationship is considered bidirectional (3): hyperglycaemia promotes periodontal tissue breakdown through advanced glycation end-products, oxidative stress and immune dysregulation, while periodontal inflammation may contribute to systemic inflammatory burden (4–7).

Type 1 diabetes (T1D) is also characterized by a pronounced inflammatory background driven by autoimmune β-cell destruction and lifelong insulin dependence (8). Although less studied, epidemiological evidence indicates that individuals with T1D have a higher prevalence and severity of periodontitis (9–12). Recent studies further suggest that T1D is associated with distinct oral microbial profiles, including shifts in community structure and enrichment in specific taxa, which may relate to metabolic control and heightened inflammatory responses (13–15).

HbA1c remains the reference marker for long-term glycaemic control, yet it does not capture short-term glucose dynamics that may be particularly relevant to inflammatory stress and interindividual metabolic heterogeneity in T1D Continuous glucose monitoring (CGM) provides complementary metrics such as Time in Range (TIR) and Time Above Range (TAR), which characterize day-to-day glycaemic exposure in T1D and offer a more granular view of metabolic complexity beyond HbA1c (16–18). Recent meta-analyses of randomized controlled trials have demonstrated that CGM use is associated with modest but significant reductions in HbA1c and mean glucose levels, along with improvements in time-in-range metrics, with reductions in TAR emerging as key contributors to overall glycaemic improvement (19). Increased TAR is recognised as a predictor of vascular complications and has also been associated with more severe periodontal outcomes (18, 20). Whether periodontitis-associated oral dysbiosis is linked to these CGM-derived short-term glycaemic metrics in T1D remains unknown.

To address this gap, we investigated whether periodontal status and salivary microbiota signatures are associated with CGM-derived indices of short-term glycaemic control (TIR/TAR) in adults with T1D. We hypothesized that periodontal status and salivary microbial ecology would vary along with CGM-derived short-term glycaemic metrics, consistent with a potential interplay between oral inflammatory conditions and day-to-day glycaemic exposure by CGM in T1D.

## 2 Materials and methods

### 2.1 Study design and ethical approval

This cross-sectional pilot study was conducted at Toulouse University Hospital in adults with T1D and was reported in accordance with STROBE guidelines. The study was approved by the French National Ethics Committee (ID-RCB: 2022-A00060-43). All participants provided written informed consent.

### 2.2 Participants

Adults (≥ 18 years) with type 1 diabetes diagnosed ≥ 2 months prior to inclusion and able to provide informed consent were eligible. Exclusion criteria were recent (≤1 month) use of antibiotics/probiotics/prebiotics, pregnancy or breastfeeding, and any legal protection status.

### 2.3 Data collection and variables

#### 2.3.1 Demographic and lifestyle data:

Age, sex, nationality, weight, height, Body Mass Index (BMI), smoking, perceived stress (on a scale from 0 to 10), and oral hygiene (tooth brushing and dental check-up frequency) were collected *via* standardized questionnaire.

#### 2.3.2 Metabolic characteristics

Glycaemic control was assessed by HbA1c and continuous glucose monitoring (CGM; FreeStyle Libre). All participants were routine users of the FreeStyle Libre CGM system as part of their standard diabetes care prior to study inclusion. CGM metrics included time in range (TIR, 70–180 mg/dL), time above range (TAR, >180 mg/dL), and time below range (TBR, <70 mg/dL), expressed as percentage of time spent in range. CGM data were analyzed over the 4 weeks preceding inclusion, with ≥ 70% valid readings required (16).

#### 2.3.3 Oral health characteristics

Examinations were performed by calibrated investigators using standardized procedures. Dental status was assessed on 28 teeth (excluding third molars) using the Decayed-Missing-Filled Teeth (DMFT) index according to World Health Organization (WHO) criteria (21). Oral hygiene was evaluated with the Plaque Index (% of teeth with visible plaque) and gingival inflammation with the Gingival Bleeding Index (% of teeth bleeding after intrasulcular stimulation with a periodontal probe) (22). Periodontal probing depth was recorded at six sites on 10 index teeth (17, 16, 11, 26, 27, 47, 46, 31, 36, and 37), with a PCP15 probe, following WHO recommendations (23). Periodontal status was defined by the Community Periodontal Index (CPI), ranging from 0 (healthy) to 4 (deep pockets), and dichotomized for analysis: CPI 1-2 (gingivitis) and CPI 3-4 (periodontitis) (24).

### 2.4 Salivary microbiota sampling and 16S rRNA sequencing

As validated in our previous studies (25, 26), unstimulated whole saliva was collected on the day of oral examination in sterile cryotubes, snap-frozen, and stored at -80 °C. Bacterial DNA was extracted using the QIAamp Cador Pathogen Mini Kit (QIAGEN, ref. 54106). The V3-V4 region of the 16S rRNA gene was sequenced (Illumina MiSeq, Vaiomer, France). Reads were processed with the FROGS pipeline and taxonomically assigned with SILVA 138.1 database.

### 2.5 Data analysis

#### 2.5.1 Statistical analyses of clinical data

Participants were classified according to the Community Periodontal Index (CPI; gingivitis: CPI 1-2; periodontitis: CPI 3-4). Continuous variables were compared using Student’s *t*-tests when distributional assumptions were met; otherwise, Mann-Whitney-Wilcoxon tests were applied. Categorical variables were compared using Fisher’s exact test. To account for potential confounding, multivariable linear regression models were fitted with TIR and TAR as dependent variables, including periodontal status (CPI 1–2 *vs*. CPI 3-4), age, sex, and perceived stress as covariates. A two-sided significance threshold of *p* < 0.05 was used. Data management was performed in Microsoft Excel^®^, and statistical analyses were conducted using Stata v13^®^ (StataCorp, TX, USA).

#### 2.5.2 Missing data handling

Missing clinical values were imputed using the MICE package in R (10 imputations; randomForest method). Analyses were performed across imputed datasets and pooled following standard multiple-imputation rules to account for uncertainty in the imputed values (27).

#### 2.5.3 Microbiota data normalization and transformation

OTU count data were treated as compositional (28). To enable downstream multivariate analyses, counts were transformed using a centered log-ratio (CLR) approach with a pseudo-count of 0.5 to handle zeros (ALDEx2 framework). CLR-transformed values were used for ordination and multivariate association analyses.

#### 2.5.4 Taxonomic composition

Taxonomic profiles were summarized at family and genus levels using stacked bar plots. For visualization, taxa with a mean relative abundance >1% were displayed individually, and remaining taxa were grouped as “Other”.

#### 2.5.5 Alpha and beta diversity analyses:

Alpha diversity (Observed, Chao1, Shannon and Simpson indices) was computed from raw count data using phyloseq. Between-group comparisons were performed using Wilcoxon rank-sum tests. Beta diversity was assessed on CLR-transformed data using principal coordinates analysis (PCoA) based on Euclidean distance. Group separation by periodontal status was tested using PERMANOVA, and confirmed by ANOSIM. Homogeneity of dispersions was evaluated using PERMDISP (vegan package). A two-sided threshold of *p* < 0.05 was applied.

#### 2.5.6 Differential abundance analysis

Differential abundance at the genus level was assessed using ANCOM-BC, which accounts for compositionality (29). Results were visualized using volcano plots (effect sizes and adjusted significance). Multiple testing correction was applied using false discovery rate (FDR); taxa were considered significant at FDR-adjusted *q* < 0.05 (and effect size thresholds where applicable).

#### 2.5.7 Associations between microbiota and clinical parameters

Cross-domain co-variation between microbial features and clinical variables (including CGM-derived metrics TIR and TAR) was explored using regularized canonical correlation analysis (RCCA; mixOmics). Results were visualized as heatmaps showing the main contributing taxa and selected clinical parameters. RCCA outputs are reported as exploratory co-variation patterns.

#### 2.5.8 Predicted functional pathway analysis

Functional profiles were inferred from the OTU table using PICRUSt2. Predicted pathway abundances were treated as compositional and analyzed on CLR-transformed data. Differential abundance of predicted pathways between periodontal groups was assessed using ANCOM-BC with FDR correction (FDR-adjusted *q* < 0.05). Associations between predicted pathways and clinical variables (including CGM-derived metrics) were explored using RCCA (mixOmics) and visualized as heatmaps of top contributing features.

#### 2.5.9 Integrative multi-block analysis

To summarize multi-domain co-variation patterns across clinical variables, bacterial taxa and predicted pathways, an integrative DIABLO analysis (block.sPLS-DA framework; mixOmics) was performed. Outputs are presented as exploratory integrative patterns and visualized using circos plots.

## 3 Results

To characterize clinical differences according to periodontal status, we compared general health, metabolic, and oral parameters between T1D patients with gingivitis and those with periodontitis (**Table 1**). Groups were comparable in sex distribution, smoking habits and oral hygiene practices. Patients with periodontitis tended to be older (46.6 years ± 12.6 *vs*. 38.8 years ± 14.9; *p* = 0.18) and reported higher stress levels (6.1 ± 2.5 *vs*. 3.8 ± 2.7; *p* = 0.03). CGM-derived short-term glycaemic metrics differed markedly between groups. The periodontitis group exhibited a significantly lower TIR (40.5% ± 18.9 *vs*. 67.7% ± 19.2; *p*= 0.004) and a higher TAR (54.4 ± 21.8% *vs.* 28.9 ± 19.2%; *p* = 0.01), whereas HbA1c and TBR did not differ significantly. In exploratory analyses, perceived stress was associated with lower TIR (r = -0.48, p = 0.01) and higher TAR (r = 0.39, p = 0.05). In multivariable models including periodontal status, age, sex, and perceived stress, severe periodontal status (CPI 3-4) remained independently associated with lower TIR (β = -21.58 ± 8.59, p = 0.021) and higher TAR (β = +21.13 ± 9.42, p = 0.037), whereas age, sex, and perceived stress were not independently associated with these outcomes. Oral examination showed higher DMFT index in the periodontitis group (10.0 ± 5.4 *vs.* 5.3 ± 4.5; *p* = 0.03) while plaque and bleeding indices were not significantly different.

**Table 1:** General, metabolic and oral characteristics of adults with type 1 diabetes according to periodontal status [gingivitis (CPI 1-2) vs periodontitis (CPI 3-4)].

Parameters	T1D subjects with gingivitis (CPI 1-2)	T1D subjects with periodontitis (CPI 3-4)	P-value
n	12	12	
Sex (number of women, %)	8 (66.7%)	9 (75%)	> 0.99
Age (years)	38.8 ± 14.9	46.6± 12.6	0.18
BMI (kg/m^2^)	26.9 ± 3.8	27.1± 5.0	0.88
Smoking (number of smokers, %)	3 (25%)	3 (25%)	> 0.99
Stress on a scale from 0 to 10 (EVA)	3.8 ± 2.7	6.1 ± 2.5	0.03*
HbA1c (%)	7.4 ± 1.10	7.8 ± 0.82	0.78
Time in Range (TIR, %)	67.67 ± 19.23	40.50 ± 18.88	0.004*
Time Above Range (TAR, %)	28.92 ± 19.16	54.42 ± 21.80	0.01*
Time Below Range (TBR, %)	2.58 ± 2,39	3.42 ± 3.15	0.59
Duration of T1D (years)	18.5 ± 17.5	11.0 ± 8.1	0.39
DMF index	5.3 ± 4.5	10.0 ± 5.4	0.03*
Numbers of Caried teeth	0.6 ± 1.0	0.8± 1.3	0.89
Number of Missing teeth	1.1 ± 1.8	3.0 ± 3.3	0.11
Number of Filled teeth	3.6 ± 3.0	6.3 ± 3.7	0.08
Plaque index (%)			0.09
< 20%	3 (25%)	0 (0%)	
20% - 50%	7 (58%)	6 (50%)	
> 50%	2 (17%)	6 (50%)	
Bleeding Index (%)	25.3± 31.1	40.8 ± 22.8	0.16
Brushing frequency			0.59
Once a day	1 (8.3%)	1 (16.7%)	
2 times a day	11 (91.7%)	9 (75%)	
3 times a day	0 (0%)	0 (0%)	
Dental check-up frequency			0.25
Less than once every 2 years	2 (18.2%)	3 (27.3%)	
Once every 2 years	2 (18.2%)	5 (45.5%)	
Once a year	5 (45.5%)	1 (9.1%)	
2 times a year	2 (18.2%)	3 (27.3%)	

**Figure 1:**
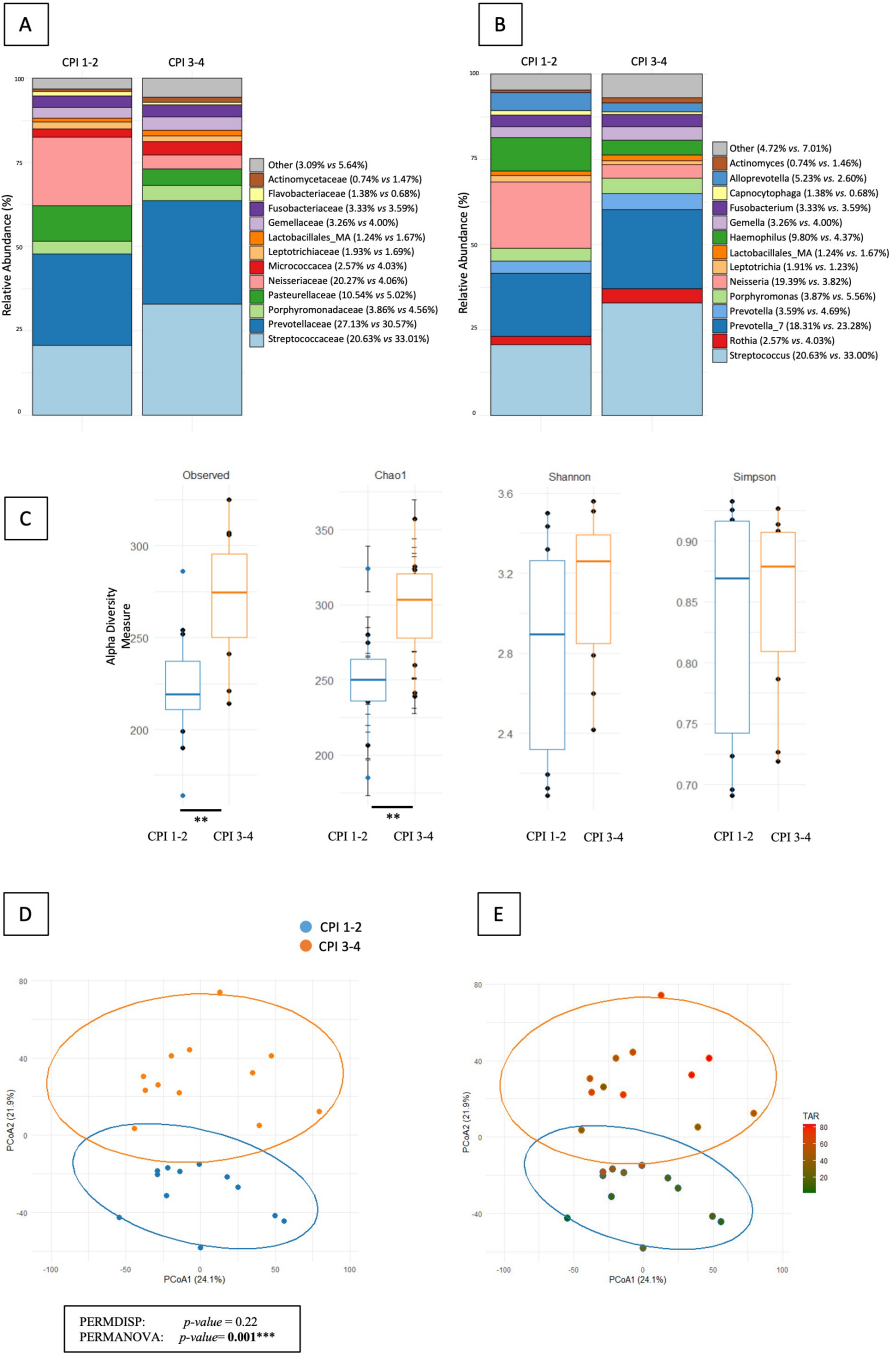
Comparison of salivary microbial profiles according to periodontal status (gingivitis (CPI 1-2) *vs* periodontitis (CPI 3-4)). **(A)** Relative abundance (%) of bacterial families in salivary microbiota. **(B)** Relative abundance (%) of bacterial genera in salivary microbiota. **(C)** Alpha diversity of salivary microbiota (Observed, Chao1, Shannon and Simpson indices). **(D)** Beta diversity of salivary microbiota assessed by principal coordinates analysis (PCoA). **(E)** Beta diversity (PCoA) of salivary microbiota with samples colour-coded according to Time Above Range (TAR, %). ** indicates a statistically significant difference with *p* < 0.01.

Salivary microbiota composition differed according to periodontal status. At the family level, the relative abundance of Neisseriaceae was significantly higher in T1D subjects with gingivitis compared to T1D subjects with periodontitis (20.27% *vs.* 4.06%; *p* = 0.03) (**Figure 1A**). At the genus level, the relative abundance of *Neisseria* was significantly higher in subjects with gingivitis compared to subjects with periodontitis (19.39% *vs.* 3.82%; *p* = 0.002) (**Figure 1B**). Alpha diversity indices reflecting richness (Observed and Chao1) were significantly higher in the periodontitis group (p = 0.004 and p = 0.008, respectively), whereas Shannon and Simpson indices did not differ, indicating unchanged evenness despite higher richness (**Figure 1C**). Beta diversity analysis revealed a distinct structuring of the salivary microbiota according to periodontal status (PERMANOVA p = 0.001), confirmed by ANOSIM (R = 0.426, p = 0.001) and independent of data dispersion (PERMDISP p = 0.22) (**Figure 1D**). Finally, PCoA plots colour-coded by TAR suggested a gradient, with higher TAR values observed in the periodontitis group (p = 0.01) (**Figure 1E**).

**Figure 2:**
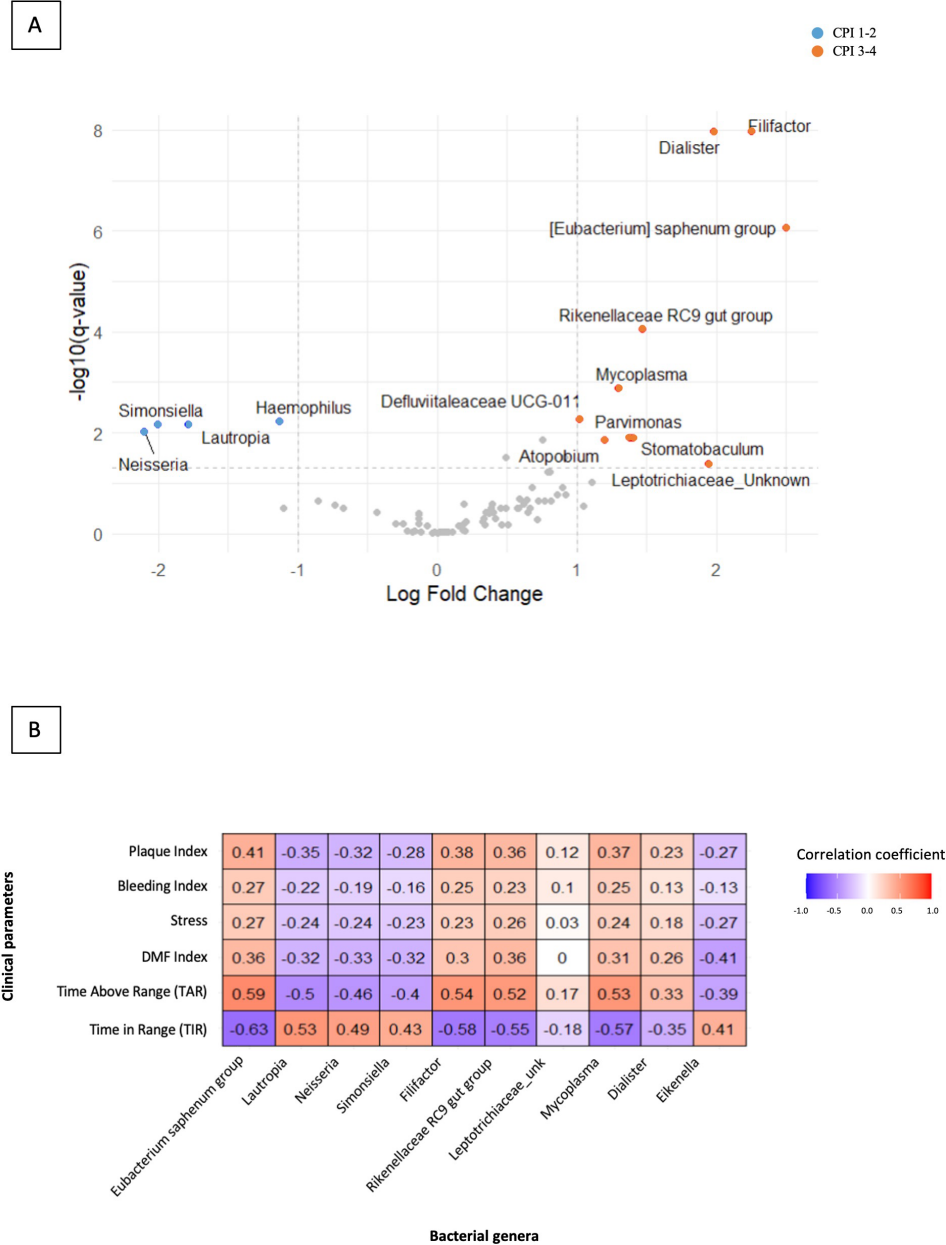
Differentially abundant salivary bacterial genera according to periodontal status and their variation with clinical parameters. **(A)** Volcano plot showing genus-level taxa differentially abundant between gingivitis (CPI 1-2) and periodontitis (CPI 3-4), as identified by ANCOM-BC. **(B)** Heatmap illustrating regularized canonical correlation analysis (RCCA) between selected bacterial genera and clinical parameters, including CGM-derived metrics.

To identify taxa differing between groups while accounting for compositionality, we performed ANCOM-BC at the genus level (**Figure 2A**). *Neisseria*, *Haemophilus*, *Lautropia*, *Simonsiella* were enriched in the gingivitis group, while *Filifactor*, *Dialister*, *Eubacterium saphenum group*, *Parvimonas*, *Mycoplasma*, *Atopobium*, *Leptotrichiaceae* and *Rikenellaceae RC9 gut group* were more abundant in periodontitis. To explore how these microbial patterns related to clinical parameters, we used regularized canonical correlation analysis (RCCA). At the genus level, *Lautropia* and *Neisseria* co-varied with higher TIR (ρ = 0.53 and 0.49 respectively) and lower TAR (ρ = -0.50 and -0.46 respectively). In contrast, *Eubacterium saphenum group* and *Filifactor*, varied along with higher TAR (ρ = 0.59 and 0.54 respectively) and lower TIR (ρ = -0.63 and -0.58 respectively) (**Figure 2B**).

**Figure 3:**
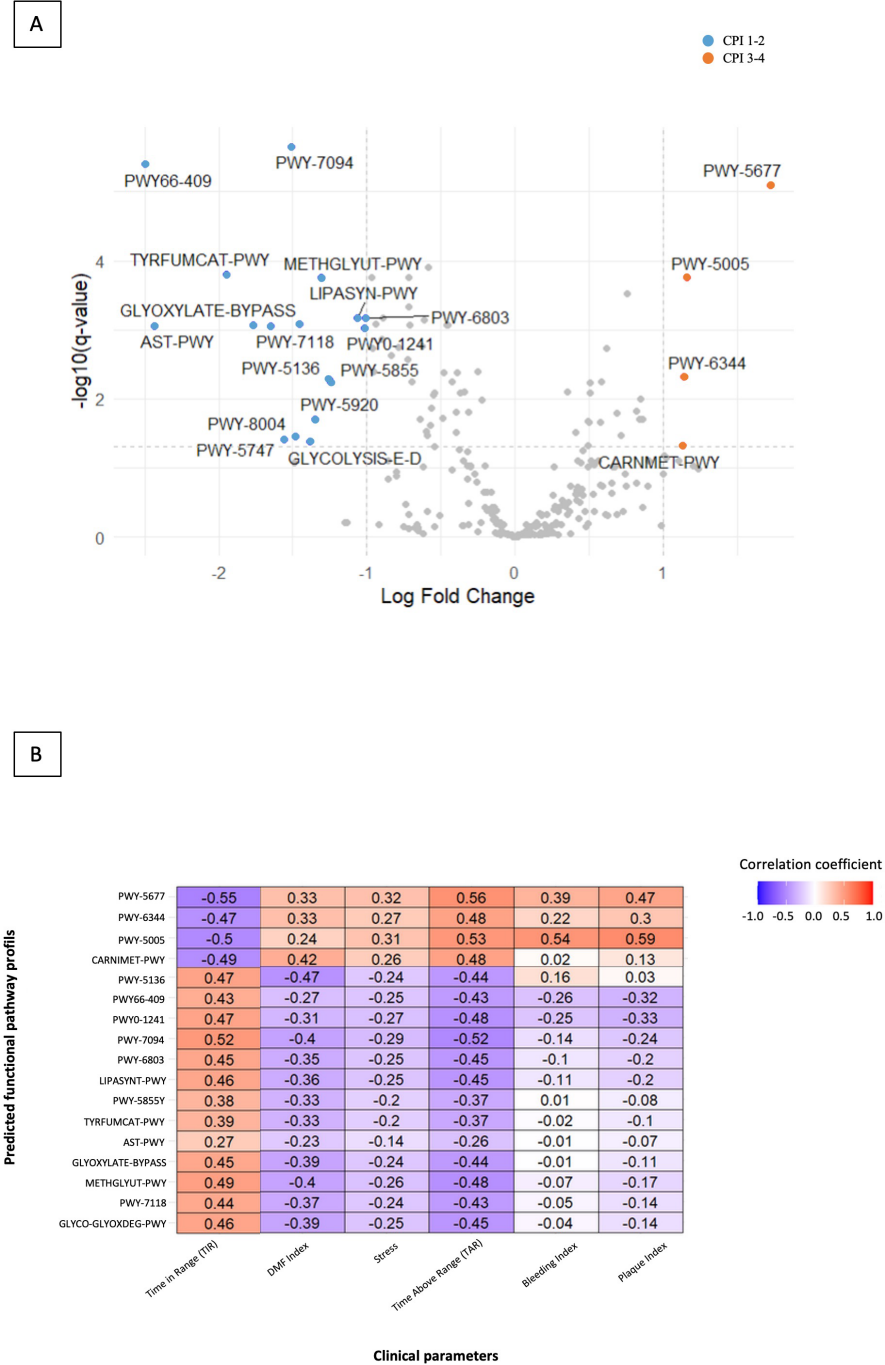
Predicted functional pathway profiles according to periodontal status and their variation with clinical parameters. **(A)** Volcano plot showing differentially abundant predicted metabolic pathways between gingivitis (CPI 1-2) and periodontitis (CPI 3-4), inferred using PICRUSt2 and identified by ANCOM-BC. **(B)** Heatmap illustrating regularized canonical correlation analysis (RCCA) between selected predicted functional pathways and clinical parameters, including CGM-derived metrics.

Predicted functional profiles (PICRUSt2) also differed according to periodontal status in patients with T1D. Volcano plot analysis revealed that gingivitis was enriched in metabolic maintenance pathways, including fatty acid salvage (PWY-7094), purine nucleotide salvage (PWY66-409), glyoxylate cycle (GLYOXYLATE-BYPASS), and methylglyoxal degradation (METHGLYUT-PWY) (**Figure 3A**). In contrast, periodontitis showed enrichment in pathways associated with fermentation and biosynthesis, such as succinate fermentation to butanoate (PWY-5677), biotin biosynthesis II (PWY-5005), L-arginine degradation XIV (PWY-6344), and L-carnitine degradation I (CARNMET-PWY). To correlate predicted pathways to clinical parameters, we performed regularized canonical correlation analysis (RCCA) (**Figure 3B**). Predicted pathways enriched in individuals with gingivitis, such as fatty acid salvage (PWY-7094) and methylglyoxal degradation (METHGLYUT-PWY), co-varied with higher TIR (ρ = 0.52 and 0.49 respectively) and lower TAR (ρ = -0.52 and -0.48 respectively). In contrast, predicted pathways enriched in individuals with periodontitis, including succinate fermentation (PWY-5677), L-arginine degradation XIV (PWY-6344) and biotin biosynthesis II (PWY-5005), co-varied with higher TAR (ρ = 0.56, 0.48, 0.53 respectively) and lower TIR (ρ = -0.55, -0.47, -0.50 respectively).

**Figure 4:**
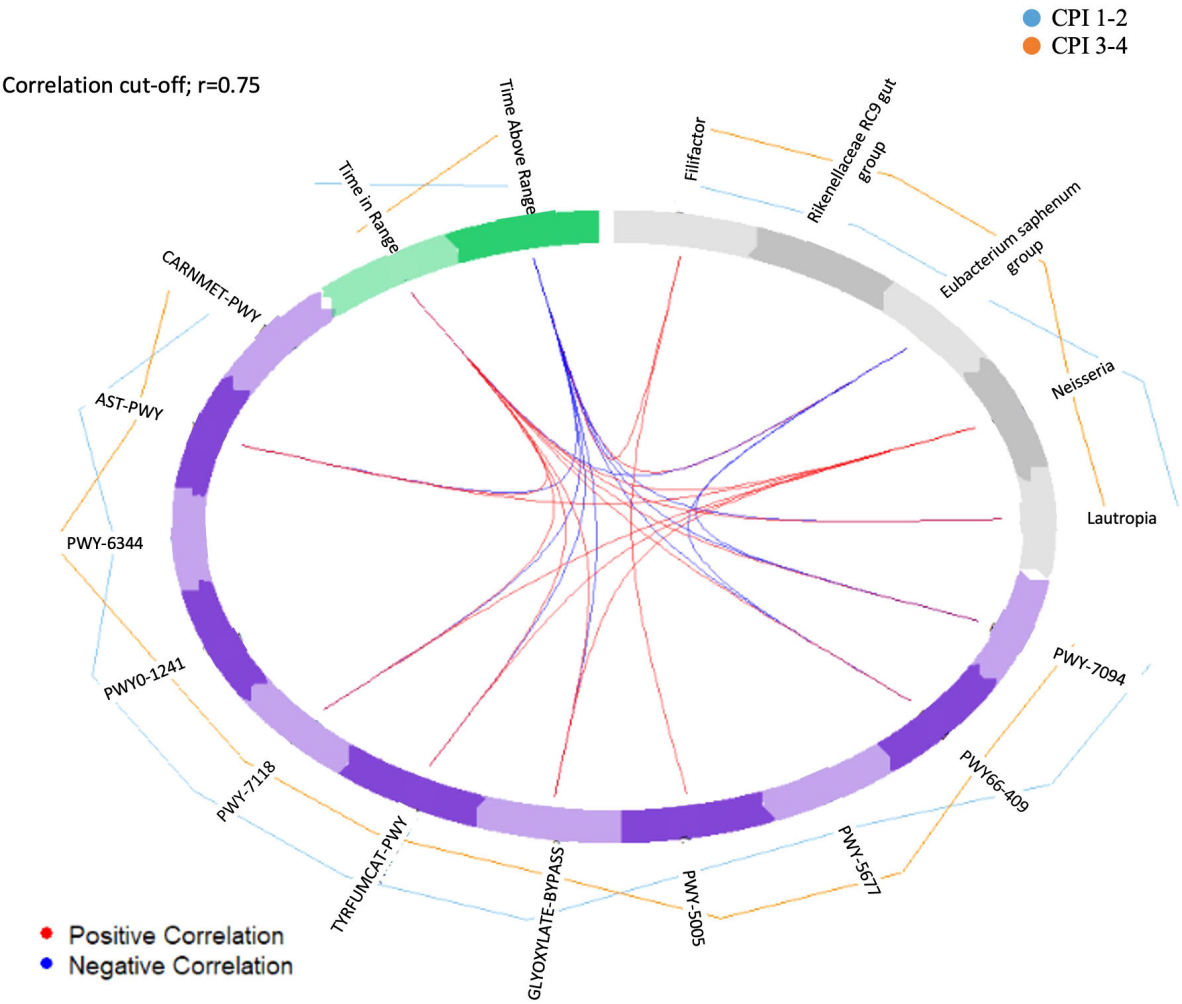
Exploratory integrative analysis of multi-domain variation between clinical parameters, salivary bacterial taxa and predicted functional pathways. Integrative DIABLO analysis (block.sPLS-DA framework) illustrating variation patterns across CGM-derived glycaemic metrics, selected bacterial genera, and predicted functional pathways. Results are visualized using a circos plot and represent exploratory integrative patterns within the cohort.

To explore the relationship between clinical parameters, bacterial taxa, and predicted metabolic pathways, we performed an integrative DIABLO analysis. The correlation between the microbial and clinical blocks on the first latent component was r = 0.75, indicating strong cross-domain association (**Figure 4**). This exploratory integration supported two contrasting profiles within the cohort. In the gingivitis group, *Neisseria* and *Lautropia* varied along with higher TIR and with predicted enrichment in metabolic maintenance/salvage pathways (including GLYOXYLATE-BYPASS and PWY66-409). Conversely, in the periodontitis group, *Filifactor* and *Eubacterium saphenum group* co-varied with higher TAR and predicted enrichment in fermentation- and biosynthesis-related pathways. DIABLO results are presented as exploratory integrative patterns (**Figure 4**).

## 4 Discussion

This pilot study addressed a clinically relevant hypothesis: whether periodontitis-related oral dysbiosis is aligned with CGM-derived short-term glycaemic metrics in adults with type 1 diabetes. Two main findings support this framework. First, periodontitis was associated with a marked deterioration in CGM metrics, lower TIR and higher TAR, despite comparable HbA1c levels. This dissociation reinforces the limitations of HbA1c as a sole marker of metabolic control in T1D, as it may conceal clinically meaningful glucose excursions, whereas CGM captures variability and exposure to hyperglycaemia that are more likely to interface with inflammatory burden and tissue-level stress. In this context, our results support the concept that CGM-derived short-term glycaemic metrics may vary along with chronic inflammatory comorbidities, such as periodontitis, for which periodontal status and oral microbiota could represent accessible biological markers. This observation is consistent with the emerging view of type 1 diabetes as a metabolically heterogeneous condition, in which CGM-derived metrics capture clinically relevant variability beyond HbA1c. Glycaemic variability may contribute to the pathophysiological link between metabolic instability and inflammatory burden in type 1 diabetes. In this population, glucose fluctuations have been associated with an increased risk of microvascular complications (30). Acute glucose excursions have also been linked to enhanced oxidative stress generation and activation of protein kinase C-dependent pathways involved in microvascular dysfunction (31). While formal variability indices were not systematically available in this cohort, CGM-derived metrics such as TIR and TAR provide complementary information on short-term glycaemic control by quantifying time spent within the target range (TIR) and above the recommended range (TAR). These metrics therefore capture aspects of dynamic glucose exposure beyond mean HbA1c levels and have been associated with complication risk in individuals with type 1 diabetes (18). Our findings are consistent with several clinical reports suggesting links between periodontal disease and glycaemic control in T1D (9, 32). Dicembrini et al. reported that clinical attachment loss correlated with indices of glucose variability but not with HbA1c (20). Jensen et al. showed that early periodontal disease markers were associated with poorer glycaemic control in young individuals with T1D (33), while Sun et al. described increased susceptibility to gingival inflammation in this population (34). Popławska-Kita et al. further reported a higher prevalence and severity of periodontitis in T1D, particularly among patients with poorer metabolic control, together with elevated systemic inflammatory mediators such as TNF-α and fibrinogen (15). These observations are compatible with a reciprocal interplay between periodontal inflammation and hyperglycaemia in T1D, although directionality and temporality require confirmation in longitudinal designs. While not tested experimentally in the present study, evidence from diabetes and hyperglycaemia literature, predominantly in T2D, suggests that glycaemic excursions may amplify AGE/RAGE signaling, oxidative stress and cytokine release. This may foster a pro-inflammatory environment that impairs periodontal tissue repair and promotes alveolar bone resorption through RANKL/OPG imbalance (1, 3, 5).

Second, periodontitis was accompanied by distinct salivary microbial and inferred functional signatures that were associated with CGM-derived metrics (TIR/TAR) within our cohort. The dissociation between increased richness (Chao1) and stable evenness (Shannon/Simpson) is compatible with ecological recruitment of accessory taxa, a pattern frequently described in inflammatory environments where niches become permissive to opportunistic colonizers. Enrichment of taxa such as *Filifactor* and the *Eubacterium saphenum group*, previously described in periodontal ecosystems was associated with higher TAR, whereas Neisseria and Lautropia were linked to higher TIR (35). Although *Filifactor alocis* is frequently reported in periodontal pathology, our taxonomic resolution was limited to the genus level, and species-specific conclusions cannot be drawn. In addition, a hyperglycemic microenvironment within the gingival crevicular fluid (GCF), as reported in individuals with diabetes (36), may alter local metabolic conditions and contribute to shifts within the periodontal niche, potentially favoring inflammation-associated anaerobic communities (37, 38). Although causality cannot be inferred from this cross-sectional dataset, a plausible interpretation is that active periodontal inflammation and barrier disruption enhance the shedding and dissemination of subgingival-associated bacteria into saliva *via* epithelial breakdown, local tissue remodeling and gingival crevicular fluid outflow, thereby imprinting saliva with a disease-relevant ecological signal (39). In this context, saliva should be considered a pooled reservoir integrating signals from multiple oral niches, and the observed salivary shifts likely reflect a spillover effect from subgingival biofilms rather than a direct site-specific assessment of the periodontal microbiota. More broadly, periodontal inflammation may represent a chronic source of immune stimulation (e.g., inflammatory mediators and microbial products) that could plausibly intersect with pathways underlying glycaemic dysregulation in T1D, particularly when assessed through CGM rather than HbA1c.

At the functional level, reconstruction of predicted functional capacity using PICRUSt2 suggested enrichment in fermentative and short-chain fatty acid–related pathways, including butyrate-associated functions. While butyrate is frequently discussed as beneficial in the gut, concentrated exposure in gingival tissues can exert cytotoxic and pro-inflammatory effects, compromise epithelial integrity, and favor a pathogenic biofilm niche (40, 41). This apparent “site paradox” likely reflects fundamental differences between oral and intestinal mucosa, including distinct host-microbe interfaces, immune tone and barrier properties such as a thinner mucin layer in gingival tissues (42–44). In this context, enrichment in butyrate-associated pathways within the salivary signature of patients with periodontitis should not be considered protective, but may instead participate in sustaining a pro-inflammatory environment and local tissue damage. By contrast, the association of *Neisseria* and *Lautropia* with more favorable CGM profiles is consistent with their frequent description as health-associated commensals. However, as the comparator group here was gingivitis rather than periodontal health, this pattern probably reflects relative preservation of ecological homeostasis rather than a return to a truly healthy baseline (45).

Several limitations warrant cautious interpretation. The modest sample size limits generalization and statistical power, and the cross-sectional design precludes causal inference and temporal ordering between periodontal status, salivary microbiota and CGM phenotypes. In addition, saliva does not provide the periodontal site-specific resolution offered by subgingival plaque. Nonetheless, saliva has clear translational advantages as a non-invasive, scalable and repeatable matrix capturing an integrated oral ecological signal across niches, making it particularly suitable for repeated sampling and future implementation in studies and in routine diabetes care (46). Overall, these results should be viewed as hypothesis-generating and provide a rationale for larger prospective studies combining CGM trajectories with deep oral phenotyping (including subgingival microbiota), host inflammatory mediators and multi-omic integration. Such designs will be required to test temporality, identify robust predictive signatures, and determine whether salivary microbial profiles can contribute to CGM-based stratification strategies addressing metabolic complexity and interindividual variability in T1D, in support of personalized patient-centered care.

## 5 Conclusion

This pilot study suggests that periodontitis-associated salivary microbiota signatures are associated with CGM-derived short-term glycaemic metrics in adults with T1D, despite similar HbA1c. These findings support the view that CGM may capture clinically relevant glycaemic exposure and fluctuations that vary along with periodontal status and oral microbial ecology. If validated in larger longitudinal cohorts, salivary profiling could complement CGM-based assessment as a rapid, non-invasive marker to support integrated periodontal and metabolic evaluation.

## Data Availability

The datasets presented in this study can be found in online repositories. The names of the repository/repositories and accession number(s) can be found below: https://www.ebi.ac.uk/ena, PRJEB98967.
